# Multimodal imaging features and genetic findings in Bietti crystalline dystrophy

**DOI:** 10.1186/s12886-020-01545-3

**Published:** 2020-08-15

**Authors:** Wei Wang, Wei Chen, Xinyue Bai, Ling Chen

**Affiliations:** 1 Department of Ophthalmology and Vision Science, the Eye & ENT Hospital, Shanghai Medical College, Fudan University, Shanghai, 200031 China; 2 Key Laboratory of Myopia of State Health Ministry (Fudan University) and Laboratory of Myopia, Chinese Academy of Medical Sciences, Shanghai, 200031 China; 3 Department of Ophthalmology, Li Ka Shing Faculty of Medicine, The University of Hong Kong, Hong Kong, China

**Keywords:** Bietti crystalline dystrophy, Multimodal imaging, CYP4V2 gene, Choroidal neovascularization

## Abstract

**Background:**

Bietti crystalline dystrophy (BCD) is a distinct entity of retinitis pigmentosa with a wide range of genotypic and phenotypic variabilities. The goal of the present study was to investigate the morphological, functional and genetic features of BCD.

**Methods:**

A full series of multimodal imaging was performed in four Chinese patients with BCD, including fundus photography, fundus autofluorescence, fundus fluorescein angiography (FFA), indocyanine green (ICG) angiography, optical coherence tomography (OCT) and microperimetry. Electrophysiological tests including full-field electroretinography (ERG) and multifocal ERG were employed. CYP4V2 gene sequencing was performed.

**Results:**

Intraretinal crystalline deposits were observed in fundus photographs in all patients. The crystals were better appreciated in infrared images. Autofluorescence imaging demonstrated multifocal patchy hypofluorescence, suggesting massive RPE atrophy. FFA and ICG angiography further confirmed atrophy of the RPE and the underlying choroidal vessels. OCT revealed disruption of the photoreceptors, RPE and the choroid. Outer retinal tubulations (ORTs) confining to the outer nuclear layer were detected in three out of four patients. Full-field ERG showed markedly diminished responses. Multifocal ERG displayed reduced central and peripheral responses in a patient with normal vision. Gene sequencing identified two deletion mutations in CYP4V2, c.802_807del and c.810delT. BCD complicated by choroidal neovascularization (CNV) was diagnosed in one patient, and intravitreal anti-vascular endothelial growth factor (VEGF) injection was given with favorable response.

**Conclusions:**

Multimodal imaging features and electrophysiological findings of BCD patients were comprehensively discussed. A novel deletion mutation, c.802_807del, in the CYP4V2 gene was reported. ORTs are important changes in the outer retina of BCD patients, further investigation of this structure may provide insights into pathology of BCD. Intravitreal anti-VEGF therapy was effective for treatment of BCD complicated by CNV.

## Background

Bietti crystalline dystrophy (BCD, MIM 210370) is an autosomal recessive inherited chorioretinal degenerative disease [1]. It is characterized by tapetoretinal degeneration with the presence of hallmark crystalline deposits in the retina and corneal limbus, retinal pigment epithelium (RPE) atrophy and choroidal sclerosis. Paralimbal corneal crystals are documented in about 1/2 ~ 1/3 cases [2]. The prevalence of BCD was 3% in a cohort of 200 patients with retinitis pigmentosa (RP), the latter is estimated to affect 2 million people worldwide [1, 3]. *CYP4V2* has been identified as the main causative gene for BCD [4, 5]. In affected individuals, the onset of disease ranges from early teens to fourth decade of life, with various presenting symptoms including paracentral scotomas (visual field constriction), nyctalopia and reduction of visual acuity.

*CYP4V2* encodes a member of the cytochromes p-450 protein family which is responsible for oxidation of substrates during fatty acid metabolism. *CYP4V2* is widely expressed in various tissues. In the eye, it is highly expressed in the choroid and the RPE while relatively less expressed in the cornea [6]. In patients with BCD, systemic abnormal lipid metabolism has been extensively discussed [6, 7]. Cells cultured from BCD patients exhibit excessive storage of triglycerides and cholesterol with reduced metabolism in transferring between lipid products when compared with normal subjects [7]. Moreover, altered serum fatty acid metabolism was detected in BCD patients [8].

The diagnosis of BCD relies mainly on the detection of characteristic intraretinal crystalline deposits together with RPE/choroidal dystrophies. Multimodal imaging has been shown to be useful in the diagnosis of BCD and differentiation from other chorioretinal degenerative diseases [9]. The aim of the present study was to describe a full spectrum of multimodal imaging features, electrophysiological and genetic findings in four Chinese patients with BCD, in an effort to provide a better understanding of the clinical features and progression of this rare disorder.

## Methods

### Patients

Four Chinese patients (Table 1) diagnosed with BCD at our clinic were included in the present study. The inclusion criterion was the presence of classical retinal crystalline deposits. Patients with other ophthalmic abnormalities such as diabetic retinal complications were excluded. This study was performed under the tenets of the Declaration of Helsinki and was approved by the Medical Ethics Committee of Eye and ENT Hospital of Fudan University.

**Table 1:** Information of four Chinese patients diagnosed with BCD

Subject No.	Age (years)	Sex	BCVA at presentation	Intraretinal crystalline deposits on fundus photography	Corneal deposits	Stage
1	32	M	20/200 OD, 20/25 OS	Yes	NF	3
2	31	M	20/20 OD, 20/20 OS	Yes	NF	3
3	66	F	20/66 OD, 20/66 OS	Yes	NF	3
4	30	M	20/25 OD, 20/130 OS	Yes	NF	3

*BCVA* best corrected visual acuity, *M* male, *F* female, *OD* right eye, *OS* left eye, *NF* not found

### Ophthalmic examinations

A comprehensive ophthalmologic examination was performed using standard protocols, including best-corrected Snellen visual acuity (BCVA), Ishihara color vision test, slit-lamp examination, fundus examination, fundus photography, autofluorescence imaging, microperimetry, fundus fluorescein angiography (FFA, TRC-50IX; Topcon Corp.,Tokyo, Japan) and indocyanine green angiography (ICGA, Spectralis HRA + OCT; Heidelberg Engineering, Heidelberg, Germany). Optical coherence tomography (OCT) images were acquired using a spectral-domain system (Heidelberg Spectralis OCT, Heidelberg Engineering, Heidelberg, Germany) as described previously [10]. Horizontal and vertical line scans centered at the fovea were obtained. Volume scans were performed to scrutinize retinal layers. Full-field electroretinogram (ERG) and multifocal ERG incorporated recommendations of the International Society for Clinical Electrophysiology of Vision (ISCEV) were employed [11, 12].

### Sequencing

Next-generation sequencing was performed to characterize mutations in the *CYP4V2* gene in our patients. Blood samples were obtained and sent to an external service to be sequenced. Signed informed consent were obtained from the patients. Direct sequencing of the entire coding regions was performed and flanking sequences of the CYP4V2 gene was determined.

## Results

**Fig. 1:**
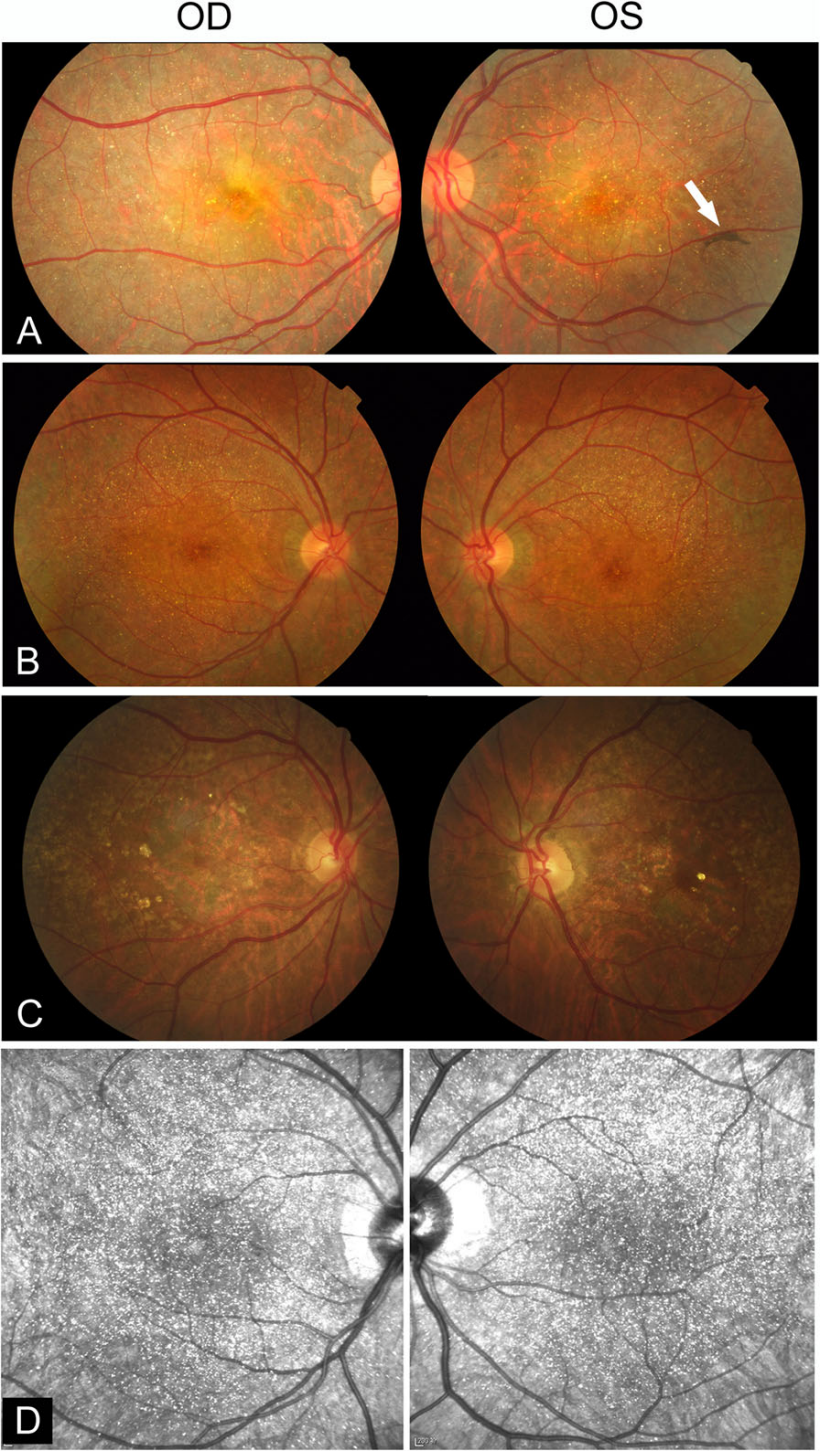
Color fundus photography and infrared images of three patients with BCD. **a** Fundus images (both eyes) of patient 1 with severe vision loss, showing retinal crystalline deposits at the posterior pole. Pigment clumping was found in the peripheral retina of the left eye (white arrow). **b** Fundus images (both eyes) of patient 2 with normal vision, showing characteristic retinal crystalline deposits throughout the posterior pole. **c** Fundus images (both eyes) of patient 3 showing crystalline deposits with RPE changes. **d** Infrared images of patient 2, wherein the intraretinal crystals are better appreciated when compared with that in the color fundus photographs

**Fig. 2:**
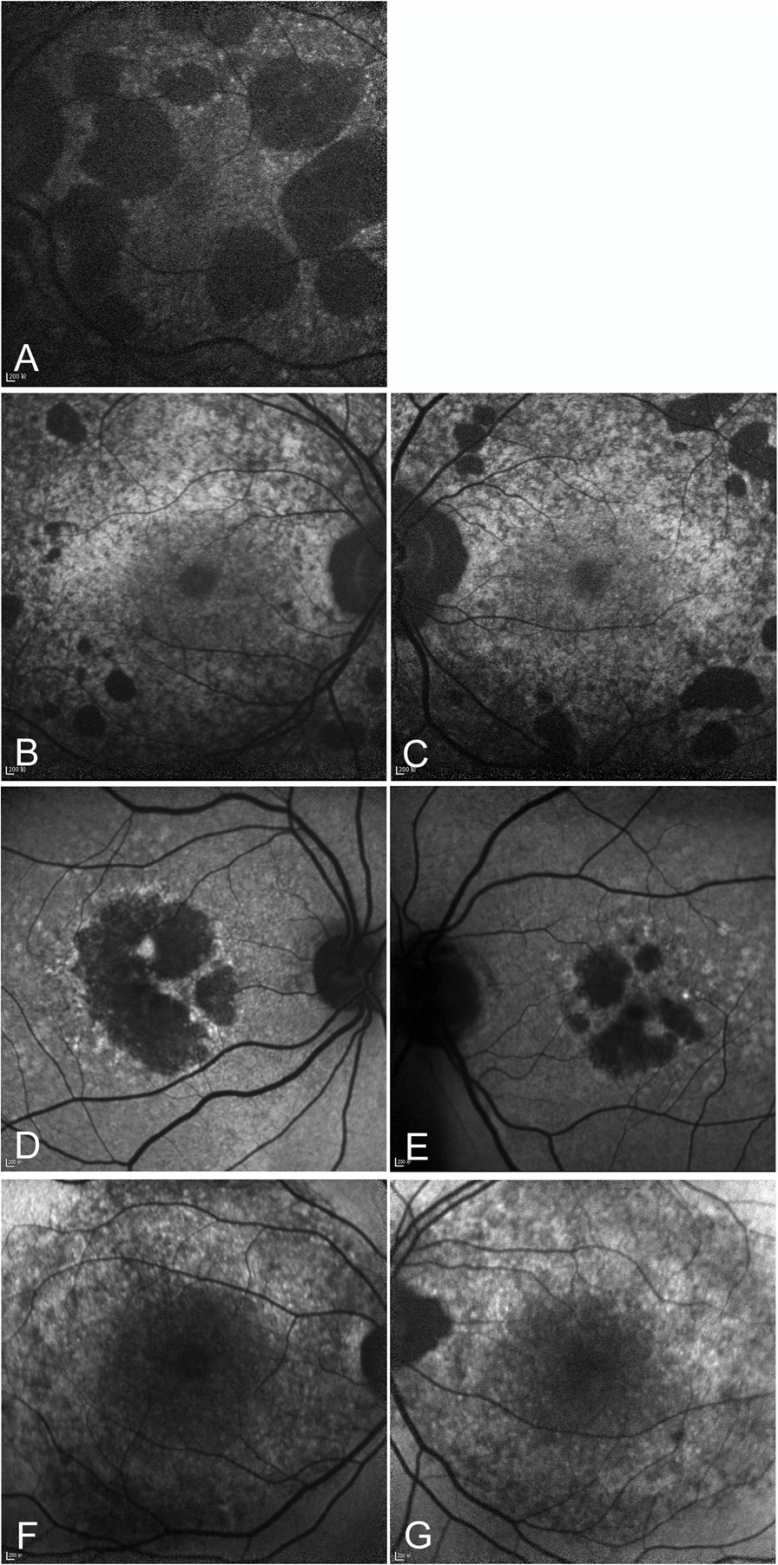
Fundus autofluorescence (AF) images of four BCD patients. **a** AF image of patient 1 (left eye) showing multiple distinct large patches of hypofluorescence in the macular area, suggesting significant retinal pigment epithelium (RPE) atrophy. The crystals are predominantly present in areas between or around the areas of RPE atrophy. **b**, **c** AF images of patient 2 (both eyes) showing small patchy hypofluorescent areas at the posterior pole with partial preservation of the foveal region. **d**, **e** AF images of patient 3 (both eyes) showing distinct patchy hypofluorescent areas in the macular area. Similarly, the crystals are present in areas between the areas of RPE atrophy and the healthy retina. **f**, **g** AF images of patient 4 showing diffuse hypofluorescence with scattered hyperfluorescence in the posterior pole

**Fig. 3:**
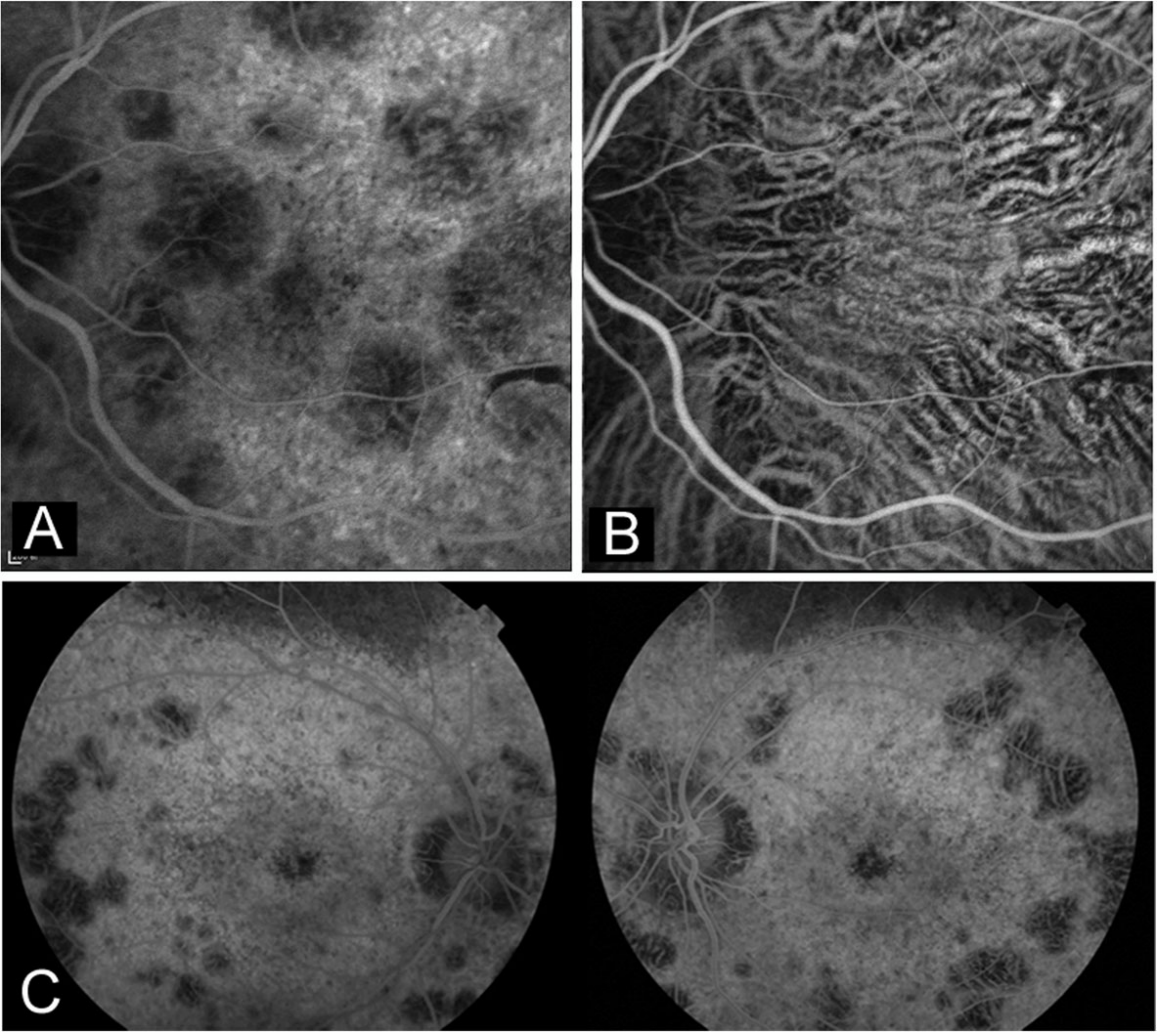
Fundus fluorescein angiography (FFA) and indocyanine green (ICG) angiography images of two BCD patients. **a** FFA image of patient 1 (left eye) showing multiple window defects corresponding to the areas of RPE atrophy at the posterior pole. **b** ICG image of patient 1 (left eye) showing altered choroidal vascular network with presence of distorted large choroidal vessels. **c** FFA image of patient 2 (both eyes) showing widespread window defects corresponding to the areas of RPE atrophy

Slit-lamp examination of the anterior segment was unremarkable in all four subjects. No corneal crystalline deposit or dystrophy was observed (Table 1). No color vision deficiency was found. In all four cases, numerous variably sized yellow-white glistening crystalline deposits were detected at the posterior poles on fundus photographs (Fig. 1) (fundus photograph of patient 4 not shown). As shown in Fig. 1, the intraretinal crystals were better appreciated in infrared images when compared with fundus photographs. Autofluorescence imaging demonstrated multifocal patchy hypofluorescent lesions interspersed with speckled hyperfluorescence, indicating atrophy of the RPE (Fig. 2). It was noted that in the atrophic patches, crystalline deposits were only occasionally seen. Instead, the crystals were predominantly found in areas between or around the areas of RPE atrophy. FFA and ICG angiography further confirmed atrophy of the RPE and alteration of the underlying choroidal vessels (Fig. 3a-c). In case 2, partial preservation of the foveal region was observed, which may explain the well-preserved visual acuity in this patient (Fig. 3c). Based on the fundus findings, all the four patients were classified into stage 3 (Table 1).

**Fig. 4:**
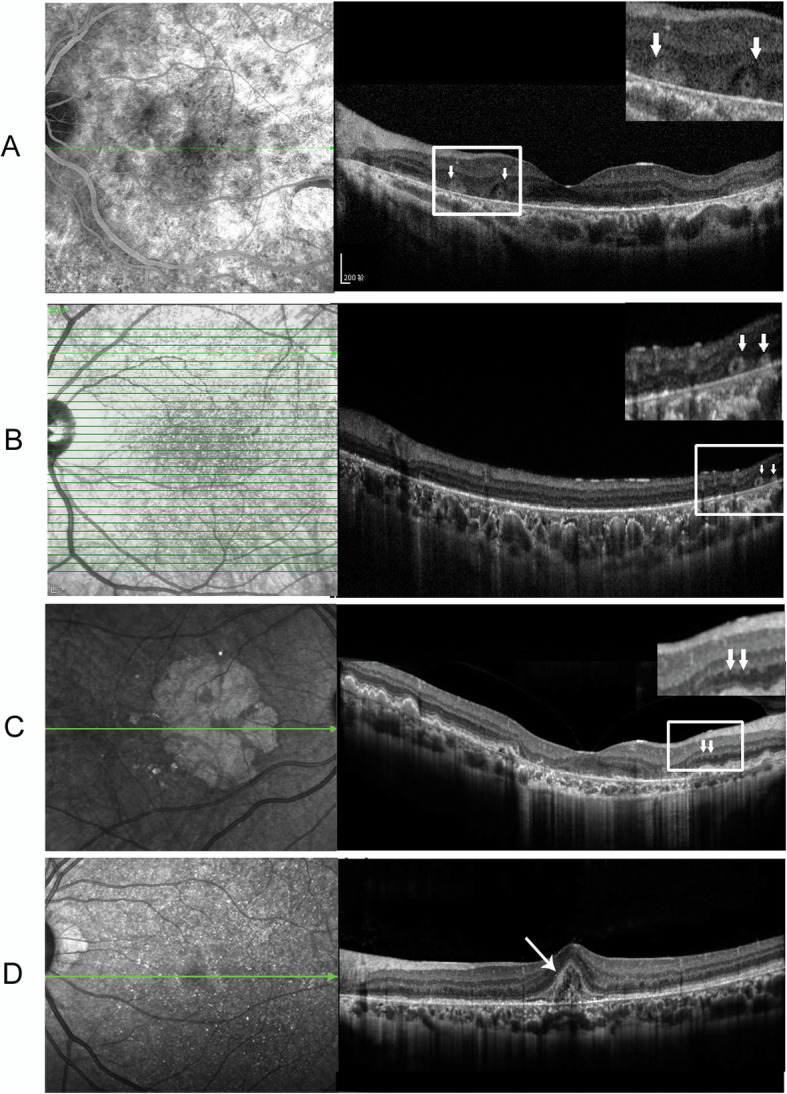
Optical coherence tomography (OCT) and corresponding fundus fluorescein angiography (FFA) images of four patients with BCD. **a**-**c** FFA and OCT images of patient 1–3. FFA images show numerous hyperreflective spots of various configurations throughout the retina as well as the overlaid retinal vessels. The outer nuclear layer (ONL) is discontinuous in the macular region. Outer retinal tubulations (ORTs) confining to the ONL are shown (**a**-**c**, white arrows). Severe disruption of the ellipsoid zone/interdigitation zone and the RPE is shown in OCT image of patient 3 (**c**). **d** FFA and OCT images of patient 4 showing extensive degeneration of the retina and the RPE/choriocapillaris complex. A hyperreflective lesion associated with remarkable RPE elevation at the foveal center is shown (white arrow)

OCT examination displayed numerous hyperreflective spots of various configurations, disruption of RPE, macular thinning and choroidal hyperreflective lesions (Fig. 4). The brightly reflective spots were present in both the deep and the superficial layers of the retina as well as the overlaid retinal vessels. Notably the hyperreflective spots located adjacent to the RPE/Bruch’s membrane correlated nicely with the crystals shown on the fundus images. The outer nuclear layer (ONL) was discontinuous in the macular region in all four patients. In case 1, extensive disruption of ONL was observed with sparing of only a small island of the foveal region (Fig. 4a). In patient 2 who was asymptomatic at the time of presentation, the neuroretina was relatively well preserved, despite the ONL loss detected by OCT (Fig. 4b). The central macular thickness (CMT) and subfoveal choroidal thickness (SFCT) of 2 patients are listed in Table 2. A marked decrease of the SFCT in patient 4 was detected (Table 2). Outer retinal tubulations (ORTs), shown as hyperreflective circular structure confining to the retinal outer nuclear layer, were detected in case 1–3 (Fig. 4a-c, white arrows). Unlike the widespread of the crystalline deposits, the ORT structures were only present in areas wherein the RPE was damaged. In the left eye of patient 4, OCT imaging revealed a hyperreflective lesion associated with remarkable RPE elevation at the foveal center (Fig. 4d, white arrow). Taken together with the leakage found in the macular area on FFA (data not shown), the diagnosis of BCD complicated by choroidal neovascularization (CNV) was considered in this patient. Intravitreal anti-vascular endothelial growth factor (VEGF) injection was given with favorable response.

**Table 2:** Values of the OCT parameters in BCD patients

Subject No.	CMT (μm)		SFCT (μm)	
	OD	OS	OD	OS
3	170	222	125	117
4	170	403	250	198

*CMT* central macular thickness, *SFCT* subfoveal choroidal thickness, *OD* right eye, *OS* left eye

**Fig. 5:**
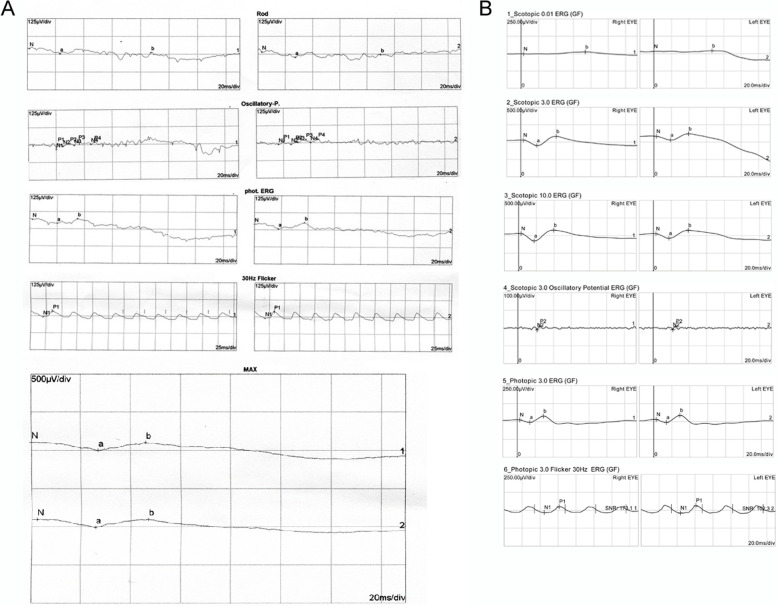
Full-field electroretinogram (ERG) results of two BCD patients. **a** Full-field ERG result of patient 1 showing non-recordable scotopic and photopic responses. **b** Full-field ERG result of patient 2 showing dramatically decreased amplitudes of photopic b-wave, scotopic a- and b-waves

**Fig. 6:**
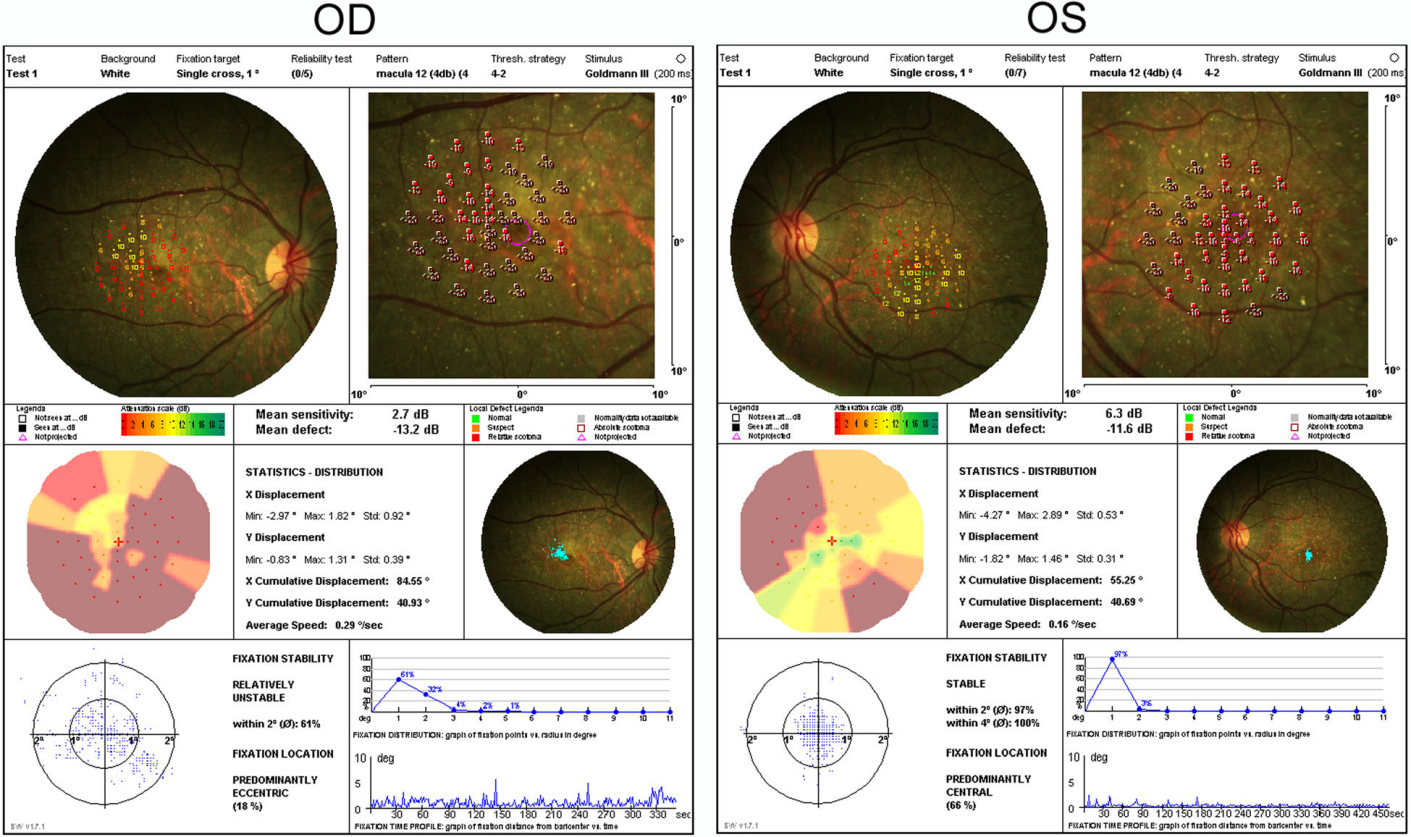
Microperimetry result of patient 1 showing bilateral central scotomas with unstable fixation in the right eye and stable fixation in the left eye

**Fig. 7:**
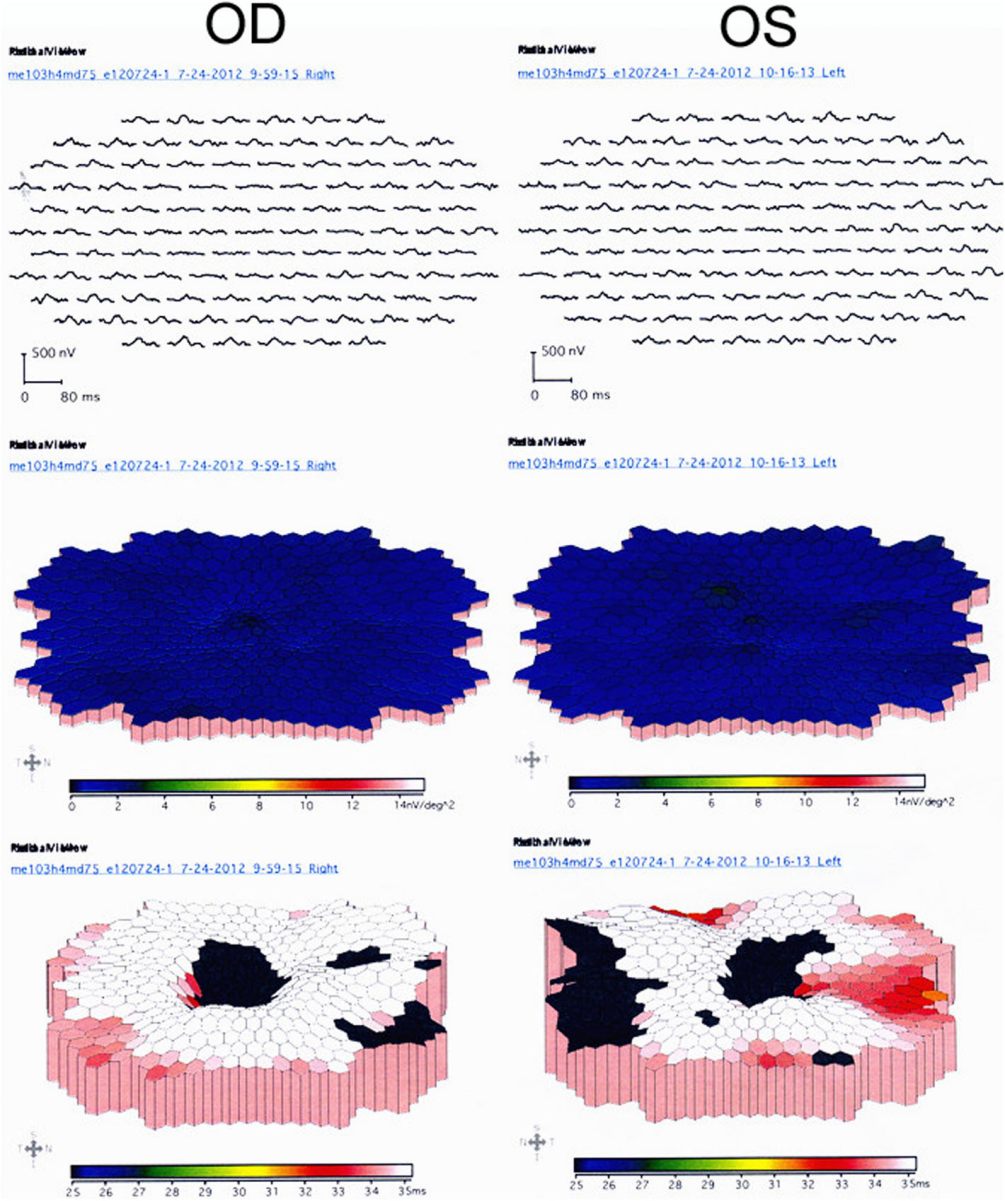
Multifocal electroretinogram (ERG) result of patient 2 showing markedly reduced response densities all over the retina with absence of the central peak

Full-field ERG showed non-recordable responses in patient 1 and 3. Rod responses were significantly reduced and the photopic wave amplitudes were severely attenuated (Fig. 5). In case 3, amplitudes of photopic b-wave, scotopic a- and b-wave were dramatically decreased. Microperimetry result of patient 1 showed bilateral central scotomas with unstable fixation in the right eye and stable fixation in the left eye corresponding with his clinical manifestations (Fig. 6). Multifocal ERG analysis in patient 2 revealed markedly reduced response densities all over the retina with absence of central peak in both eyes (Fig. 7).

Molecular analysis of the CYP4V2 gene revealed two homozygous deletion mutations in patient 1: c.802_807del (p.268_269del) and c.810delT (p.A270fs). The c.802_807del mutation has not been published in the Human Gene Mutation Database (HGMD). This is the first report of this deletion mutation in BCD patient. c.810delT deletion is a known frameshift mutation and has been reported in BCD patients as a disease-causing mutation [13].

## Discussion

The diagnosis of BCD was made in our patients based on the characteristic presentation, including the presence of yellowish-white reflective intraretinal crystals, widespread retinal and choroidal thinning, and choriocapillary atrophy. It is noted that none of our patients showed corneal changes such as crystalline deposits or atrophy. This is consistent with previous report of high prevalence of pure retinal involvement in Asians [14, 15]. BCD is a slowly progressive disease. According to the clinical evaluation method described by Yuzawa et al. [16], the disease progression of BCD can be divided into three stages: early, intermediate and advanced stages. In the early stage, the retinal crystals are disseminated throughout the posterior pole, and the chorioretinal atrophy tends to be limited to the posterior pole as well. In the intermediate stage, the crystals diminish in number at the posterior pole with the chorioretinal atrophy expanding centrifugally to the equator. In the advanced stage, extensive chorioretinal atrophy is developed, crystals may be occasionally seen or absent. Considering that the intraretinal crystals become less apparent as disease progresses, the diagnosis of BCD becomes more challenging in the late stage of disease.

The origin and nature of the crystalline deposits are unclear, yet their role in RPE and retinal degeneration remains to be determined. In our study, intraretinal crystalline deposits with a range of severity were detected by fundus examination. In our observation, the crystalline deposits in the retina were only present between the atrophic areas. In other words, the crystals were located selectively in the relatively “healthy” areas of the retina as reported [17, 18]. Gocho et al. proposed that the retinal crystals may have a pro-survival role, as they found that the cone photoreceptors located over the crystalline deposits appeared healthier when compared with those in the atrophic areas [18]. Infrared images tended to be superior to fundus photographys in displaying intraretinal crystals [9, 19]. This finding is consistent with a previous report, in which the near-infrared reflectance (NIR) imaging demonstrated 100% sensitivity and specificity for differential diagnosis of BCD [9].

OCT imaging is a useful method in specifying the anatomical location of the crystals in the retinal layers. By analyzing the crystals on fundus photographs/infrared images and the hyperreflective spots in OCT images, many authors proposed that the crystals might be located in the RPE/Bruch’s membrane complex [19, 20]. This is also confirmed in our study. Moreover, measurement of OCT parameters such as CMT and SFCT (Table 2) may provide additional information on disease severity and progression [10]. ORT is another important morphologic change found in our patients. As revealed by OCT imaging, these circular structures were confined to the outer nuclear layer/photoreceptor layer of the retina. ORTs have been described in BCD as well as other outer retinal degenerative disorders such as age-related macular degeneration (AMD) and RP. ORTs were first described by Zweifel et al. in 2009 and were speculated to be a misguided reparative attempt of degenerating photoreceptors [21]. Zweifel and colleagues hypothesized that after sublethal injury (e.g. through RPE degeneration), tight junctions of the photoreceptors were disrupted, followed by rearrangement of the photoreceptors in a tubular fashion [21]. This may partially explain our observation that ORTs were only present in areas where RPE was broken and in lesions where the RPE/choriocapillaris complex was relatively more diminished. Currently, the presence of ORTs is considered as an indicator of ongoing active degeneration. Clinical studies showed that ORTs were associated with poor visual prognosis [22–24]. Moreover, when compared with other degenerative retinal diseases, ORTs are more frequently detected in BCD [17]. In our study, ORTs were detected in three out of four patients. Further investigation of this characteristic structure may help provide a more comprehensive understanding of the pathology of BCD.

Electrophysiological test is helpful to determine the extent of retinal function loss in BCD patients. Full-field ERG, which reflects function of the entire retina, can show varying degrees of rod and cone dysfunction depending on the stages of disease progression [25, 26]. However, atypical case was reported, wherein patient with classical RPE/choriocapillaris lesions showed normal ERG responses [27]. In our study, full field ERG showed non-recordable responses in case 1 and severely reduced a- and b-wave amplitudes in case 3, suggesting severe panretinal dysfunction in both patients. These findings were in accordance with their advanced clinical features and OCT changes. Different from full-field ERG technique, multifocal ERG is a newer electrophysiological method that can record abnormal responses from different retinal regions. In case 2, multifocal ERG was employed to measure the function of the residual foveal region. However, the result demonstrated dramatically diminished central responses which corresponded poorly with the patient’s good visual acuity.

Disease-causing mutations in *CYP4V2* have been widely studied in patients with BCD, including missense, nonsense, insertion or deletion mutations etc. Most mutations reported in BCD patients are missense mutations. In the present study, we identified two homozygous deletion mutations in the *CYP4V2* gene, c.802-807del and c.810delT in patient 1. The c.802-807del deletion has not been reported before. Although no explicit genotype-phenotype correlation has been determined in BCD patients, studies have suggested that genetic deletions might be associated with a more severe disease and worse visual acuity when compared with relatively mild mutations [19, 28]. Further investigation of the c.802-807del deletion may elaborate its functional implications in BCD. And this may also facilitate our understanding of the difference in disease severity and clinical manifestations between patient 1 and 2 in our study. Additionally, a recent study revealed that the *CYP4V2* gene mutation may have a causal effect on choroidal vascular damage thus contributing to visual impairment in BCD patient [6]. Moreover, other factors such as diet and environment may also play roles in the pathogenesis and contribute to the phenotypic variations.

In addition to the classical features described by conventional methods, more characteristics of BCD have been reported recently with the utilization of optical coherence tomography angiography (OCTA). Miyata et al. reported a significant reduction in choriocapillaris blood flow in BCD patients when compared with that in the healthy subjects by using OCTA [29]. Similarly, Ipek et al. reported a marked decrease of choroidal flow in a BCD patient at 10-year follow-up [30]. Furthermore, a diminshed outer choroidal vascular area was detected in the BCD patients by analyzing en face images obtained with swept-source optical coherence tomography (SS-OCT) [31]. With increasing use of OCTA method, further analysis of choroidal vasculature changes in BCD patients may provide new insights into the etiology and evolution of the disease. Lacking of OCTA result is a limitation of the present study. Utilization of OCTA in follow-up examinations in these patients may be helpful in assessing disease progression.

CNV is a complication rarely reported in BCD patients. Although breakdown of Bruch’s membrane is known to be present, the explicit pathogenesis of CNV in BCD patients is unclear. In common with other reported BCD cases complicated by CNV, the CNV lesion observed in our patient appeared to confine within the foveal/parafoveal retina. Therefore, the development of CNV is not likely a direct consequence of the intraretinal crystals, as the latter tend to be scattered throughout the posterior pole. Fuerst et al. demonstrated that CNV in BCD patients may be attributed to the dysfunction of RPE and abnormalities of photoreceptor outer segment-RPE interface at the fovea and parafovea [32]. Anti-VEGF therapy has been shown to be effective to induce CNV regression and vision improvement in BCD [33, 34]. In our case, given the presence of macular hemorrhage, anti-VEGF injection was administered intravitreally with favorable response.

## Conclusions

BCD is a distinct entity of retinitis pigmentosa with a wide range of genotypic and phenotypic variabilities. Our study provided comprehensive information regarding this rare condition from multimodal imaging, electrophysiological test and genetic analysis. We identified two homozygous mutations, c.802_807del and c.810delT, in our patient. This is the first report of c.802_807del mutation in patient with BCD. ORTs are important morphological changes frequently detected in BCD patients, further investigation of this structure may provide insights into pathology of BCD. Intravitreal anti-VEGF therapy is effective for treatment of BCD complicated by CNV.

## Data Availability

All data supporting the current study are available from the corresponding author on reasonable request.

## Abbreviations

AMD: Age-related macular degeneration
Anti-VEGF: Anti-vascular endothelial growth factor
BCD: Bietti crystalline dystrophy
BCVA: Best-corrected Snellen visual acuity
CMT: Central macular thickness
CNV: Choroidal neovascularization
ERG: Electroretinography
FFA: Fluorescent fundus angiography
ICG: Indocyanine green
ISCEV: International Society for Clinical Electrophysiology of Vision
OCT: Optical coherence tomography
ONL: Outer nuclear layer
ORT: Outer retinal tubulation
RP: Retinitis pigmentosa
RPE: Retinal pigment epithelium
SFCT: Subfoveal choroidal thickness
SS-OCT: Swept-source optical coherence tomography
